# Editorial: Diet-sleep interaction on cardiometabolic health

**DOI:** 10.3389/fpubh.2023.1190615

**Published:** 2023-04-05

**Authors:** Vicky Wai-ki Chan, Kenneth Ka-hei Lo

**Affiliations:** Department of Food Science and Nutrition, The Hong Kong Polytechnic University, Hung Hom, Hong Kong SAR, China

**Keywords:** cardiometabolic, dietary pattern, Mediterranean diet, nocturnal metabolism, sleep duration, sleep quality

Poor dietary and sleep habits are well-established risk factors for adverse cardiometabolic health, including obesity, metabolic syndrome, diabetes, and cardiovascular diseases (1, 2). Furthermore, emerging physiological evidence has suggested the bi-directional relationship between diet and sleep. While some dietary factors may increase the availability of tryptophan or enhance the synthesis of serotonin and melatonin (3, 4), poor sleep may relate to unhealthy diet by hormone dysregulation, increased sensitivity to food reward, greater time opportunity, and impaired decision making (5). However, diet and sleep are usually treated as separate lifestyle factors for evaluating the association with health outcomes. Without targeting both lifestyle factors simultaneously, researchers may overlook their additive benefits in cardiometabolic health, particularly when implementing lifestyle interventions. Therefore, the goal of this Research Topic is to summarize the recent advances on the interactive effects of diet (e.g., nutrients, food groups, or dietary patterns) and sleep (e.g., sleep duration, quality, or disorder) on cardiometabolic health.

In the first article, Mesas et al. conducted a cross-sectional study to investigate whether blood pressure level was associated with adherence to the Mediterranean diet and habit of regular siestas in 698 Spanish adolescents. They found that adolescents who had high adherence to the Mediterranean diet or took a siesta regularly were less likely to have high blood pressure. However, in combination of both behaviors, an additional potential benefit was not demonstrated. The Mediterranean diet, which emphasized the consumption of fresh fruits, vegetables, nuts, whole grains, and healthy fats, was suggested to have cardiocirculatory benefits such as reducing inflammatory markers levels, enhancing cardiorespiratory fitness, and maintaining normal blood pressure (6, 7). The relationship between siestas and blood pressure level had not yet come to a consensus. However, siestas were suggested to be associated with positive mood and reduced inflammatory biomarker levels, which was ultimately beneficial to blood pressure control (8). The findings suggested that early adoption of both behaviors could be a strategy in hypertension prevention throughout adulthood.

For the second article, Kanagasabai et al. explored the potential mediating roles of C-reactive protein (CRP), gamma glutamyl transferase (GGT), and micronutrient antioxidants (bilirubin, carotenoids, uric acid, vitamins A, C, D, and E) to the relationships between sleep-fasting insulin concentration and glycated hemoglobin (HbA1c) in 1,946 US adult population. They found that GGT, carotenoids, uric acid, vitamins C and D had significant contributions to the relationship between sleep duration and fasting insulin concentration, while CRP, bilirubin, and vitamin C had significant contributions to the relationship between sleep quality and fasting insulin concentration. Poor sleep quality was suggested to impact several biochemical analytes associated with oxidative stress and inflammation. This would activate inflammatory pathways and drive the development of whole-body insulin resistance, especially in middle-aged women (9). This study revealed the significant contributions of CRP, GGT, bilirubin, carotenoids, uric acid, vitamins C and D to the association between sleep and fasting insulin concentration.

For the third article, Udeh-Momoh et al. examined the relationship between health, lifestyle, and psycho-social factors and sleep quality in 5,558 UK older adults during the first pandemic phase. They observed that lifestyle factors (i.e., higher alcohol consumption and unhealthy diet) and psychosocial factors (i.e., higher anxiety levels and depressive symptoms) were associated with poor sleep quality. Also, positive association was observed in participants who were younger, females, with multi-morbidity, clinically extremely vulnerable (CEV) to coronavirus, with higher BMI, solitary, suffering from arthritis, chronic obstructive pulmonary disease, and mental health disorders. Their findings further highlighted that sleep quality was more disrupted in CEV males with higher level of anxiety while in CEV females, sleep quality was affected more by dietary intervention. This study reinforced the significance in assessing the role of risk factors while tailoring health intervention or policy on sleep quality.

As the last article of this Research Topic, Mantantzis et al. performed a review of the effects of dietary carbohydrates and proteins on sleep modulation, nocturnal metabolism regulation and sleep-related health benefits. They concluded that diets with lower carbohydrate quality index were associated with higher prevalence of subjective sleep complaints while diets rich in protein could enhance sleep quality by reducing awakenings and sleep fragmentation. They also identified the important role of meal timing and carbohydrate composition of diet on sleep *via* nocturnal metabolism regulation, where having high-carbohydrate diets just before sleep could reduce slow wave sleep but 4 h before could facilitate sleep onset. Although high dietary protein and carbohydrate intake could boost serotonin and melatonin synthesis and promote sleep onset, the review argued that the accompanied hyperinsulinemia and counter regulatory hormonal responses would increase sleep fragmentation and thus lower sleep quality. They also disclosed the significant gap in identifying the relationship between nocturnal glycemia and next-day benefits in healthy community.

As demonstrated from the four papers, the pathways that connect diet, sleep, and cardiometabolic can be complex and worth further investigations in mechanistic studies, large-scale prospective cohorts or intervention studies. While the potential physiological mechanisms of the bi-directional diet-sleep relationship have been established from basic science research, epidemiological studies are required to quantify the relative importance of each biological pathway. This can be done by statistical methods such as mediation analysis, pathway analysis, or more sophisticated methods in machine learning (10). With the advancement in research methods, that will provide us with more solid evidence to develop effective dietary strategies that can alleviate the adverse effect of poor sleep quality or circadian disruption on cardiometabolic health, such as aligning meal timing with individuals' chronotype, and having the appropriate dietary habits during shift work. The upcoming research work will also be in line with the 2020–2030 Strategic Plan for NIH Nutrition Research, which is to determine the health benefits and mechanisms of time-based dietary patterns, in other words, what and when to eat to improve health.

## Author contributions

Both authors listed have made a substantial, direct, and intellectual contribution to the work and approved it for publication.

## References

[1] Jansen EC Dunietz GL Tsimpanouli ME Guyer HM Shannon C Hershner SD. Sleep, diet, and cardiometabolic health investigations: A systematic review of analytic strategies. Curr Nutr Rep. (2018) 7:235–58. 10.1007/s13668-018-0240-3 30187293 PMC6237660

[2] Lo K Woo B Wong M Tam W. Subjective sleep quality, blood pressure, and hypertension: A meta-analysis. J Clin Hypertens. (2018) 20:592–605. 10.1111/jch.13220 29457339 PMC8031314

[3] Dashti HS Scheer FA Jacques PF Lamon-Fava S Ordovas JM. Short sleep duration and dietary intake: Epidemiologic evidence, mechanisms, and health implications. Adv Nutr. (2015) 6:648–59. 10.3945/an.115.008623 26567190 PMC4642416

[4] Lo K Huang YQ Liu L Yu YL Chen CL Huang JY. Serum Vitamin D, Sleep pattern and cardiometabolic diseases: Findings from the National Health and Nutrition Examination Survey. Diabetes Metab Syndr Obes. (2020) 13:1661–8. 10.2147/DMSO.S256133 32523366 PMC7234964

[5] Fatima Y Doi SA Mamun AA. Longitudinal impact of sleep on overweight and obesity in children and adolescents: A systematic review and bias-adjusted meta-analysis. Obes Rev. (2015) 16:137–49. 10.1111/obr.12245 25589359

[6] Santi-Cano MJ Novalbos-Ruiz JP Bernal-Jiménez M Bibiloni MDM Tur JA Rodriguez Martin A. Association of adherence to specific mediterranean diet components and cardiorespiratory fitness in young adults. Nutrients. (2020) 12:776. 10.3390/nu12030776 32183454 PMC7146290

[7] Sureda A Bibiloni MDM Julibert A Bouzas C Argelich E Llompart I. Adherence to the mediterranean diet and inflammatory markers. Nutrients. (2018) 10:62. 10.3390/nu10010062 29320413 PMC5793290

[8] Faraut B Andrillon T Vecchierini MF Leger D. Napping: A public health issue. From epidemiological to laboratory studies. Sleep Med Rev. (2017) 35:85–100. 10.1016/j.smrv.2016.09.002 27751677

[9] Kim TH Carroll JE An SK Seeman TE Namkoong K Lee E. Associations between actigraphy-assessed sleep, inflammatory markers, and insulin resistance in the Midlife Development in the United States (MIDUS) study. Sleep Med. (2016) 27–8:72–9. 10.1016/j.sleep.2016.07.023 27938923 PMC5206915

[10] Zhao Y Naumova EN Bobb JF Claus Henn B Singh GM. Joint associations of multiple dietary components with cardiovascular disease risk: A machine-learning approach. Am J Epidemiol. (2021) 190:1353–65. 10.1093/aje/kwab004 33521815 PMC8245893

